# Supplementary material for: Cost-effectiveness and benefits of perinatal health interventions in high-income settings: A protocol for a systematic review of economic evaluations
Source: PLoS One. 2024 Jul 2;19(7):e0306557. doi: 10.1371/journal.pone.0306557 (PMC11218939; doi:10.1371/journal.pone.0306557)
Supplement: Attachment — Submitted filename: Response to reviewers_Haile et al.,.docx [file pone.0306557.s003.docx]

**21^st^ May 2024**

Re: Cost-effectiveness and benefits of perinatal health interventions in high-income settings: a protocol for a systematic review of economic evaluations (**Manuscript #:** PONE-D-23-42050R1)

Prof Miquel Vall-llosera Camps

*Senior Staff Editor, PLOS ONE*

**Re: Response to reviewers’ comments**

Dear Professor Miquel,

We are pleased to submit our revised version protocol entitled Cost-effectiveness and benefits of perinatal health interventions in high-income settings: a protocol for a systematic review of economic evaluations (manuscript ID: PONE-D-23-42050R1). We greatly appreciate the reviewers' comments and feedback, which will enhance our upcoming review.

We have addressed all the comments, incorporated the suggestions, responded to them point-by-point, revised the protocol accordingly, and presented the revisions in track changes. At this stage, we hope that you find the revised protocol acceptable for publication.

Thank you for your time and consideration.

Kind regards,

Tsegaye G. Haile (MSc, MPH, PhD Candidate), corresponding author

Dr Gizachew A. Tessema, Dr Lucas Hertzog, Dr Elizabeth Newnham, Dr Berihun Dachew, Dr Marshall Makate

**Response to reviewers’ comments**

**Reviewer 1**

The authors have made a substantial effort to improve their manuscript and have taken the suggestions provided by the reviewers to heart.

**Q1:** I have one comment still with regard to the definition of the comparators give the updated scope of the review: the authors have chosen to not include partial economic evaluations in their planned review anymore, which I feel is a sensible decision. However, since a full economic evaluation would always include a comparative element (see also the clear definitions in this paper: https://www.ncbi.nlm.nih.gov/pmc/articles/PMC8424074/ ) - the fourth comparator on p10 of the clean manuscript, stating 'No comparators: Economic evaluations focusing solely on the costs of perinatal health interventions without specific comparators.' - is not a valid comparison here - this could only be a relevant thing to study when partial economic evaluations would be included. So this 4th option should be removed from the list.

**Response:** Dear reviewer, thank you so much for reviewing our second version protocol. Your comments and feedback greatly improve our protocol and for our planned actual review. We have removed the fourth comparator 'No comparators: Economic evaluations focusing solely on the costs of perinatal health interventions without specific comparators' as we will include the full economic evaluations. We have also updated the search terms to focus solely on the full economic evaluations. The change has been found on page pages 8 and 10.

**Q2.** Last remark: given that scope and search terms have been updated, will this also be aligned in the prospero protocol, that is, will prospero be updated accordingly?

**Response:** Thanks for the remarks. We have mentioned the updates on the PROSPERO registrations (PROSPERO registration # CRD42023432232).

**Reviewer 3**

Recommendations: This review is expected to notably affect healthcare practices by summarizing the financial impacts and benefits of perinatal interventions, aiding in the optimization of strategies to avert negative perinatal outcomes.

Reply: Dear reviewer, thank you very much for highlighting the importance of our upcoming review.
